# Development of poly-D-mannose-stabilized silver nanoparticles exhibiting antibacterial and antibiofilm properties

**DOI:** 10.1128/aem.01131-25

**Published:** 2025-10-14

**Authors:** Dandan Tian, Xiaoqing Xu, Fengjiao Zhang, Weifeng Chen, Huang Zhang, Bo Shi

**Affiliations:** 1 School of Food and Biological Engineering, Henan University of Animal Husbandry and Economy 146228 , Zhengzhou, China; 2 Feed Research Institute, Chinese Academy of Agricultural Sciences 471744 , Beijing, China; Anses, Maisons-Alfort Laboratory for Food Safety, Maisons-Alfort, France

**Keywords:** antibiofilm, antimicrobial, silver nanoparticles, poly-D-mannose

## Abstract

In this study, previously synthesized poly-D-mannose (PM) served as the stabilizing agent for the production of silver nanoparticles (designated PM-AgNPs), which were subsequently assessed for their antibacterial and antibiofilm capabilities against foodborne pathogens. The PM-AgNPs were successfully synthesized, exhibiting a distinct surface plasmon resonance absorption peak at 400 nm. Fourier transform infrared analysis revealed the stabilization mechanism of silver nanoparticles by PM, involving the formation of O–H⋯Ag hydrogen bonds and C–O⋯Ag coordination bonds. Both transmission electron microscopy and dynamic light scattering measurements confirmed the spherical morphology of PM-AgNPs, with particle sizes ranging from 2 to 18 nm and a zeta potential of −24.7 mV. Furthermore, energy-dispersive X-ray spectroscopy analysis disclosed the elemental composition of PM-AgNPs to be C:O:Ag in a ratio of 39.32:37.93:22.75. To evaluate the antibacterial and antibiofilm efficacy of PM-AgNPs against selected pathogenic bacteria and their biofilms, the broth microdilution method and 3-(4,5-dimethylthiazol-2-yl)-2,5-diphenyltetrazolium bromide staining assay were employed, respectively. The inhibitory effect of PM-AgNPs on five pathogenic bacteria was found to be in the following order: *Pseudomonas aeruginosa* > *Escherichia coli* O157:H7 > *Salmonella* Typhimurium > *Staphylococcus aureus* > *Bacillus cereus*, with corresponding minimum inhibitory concentrations of 23.8 µg/mL, 95 µg/mL, 190 µg/mL, 380 µg/mL, and 380 µg/mL, respectively. The efficacy of PM-AgNPs in suppressing the biofilms of the five aforementioned pathogenic bacteria showed a ranking of *S. aureus* > *S.* Typhimurium > *E. coli* O157:H7 > *B. cereus* > *P. aeruginosa*. In summary, PM-AgNPs exhibit promising potential as an antibacterial agent in the food industry.

**IMPORTANCE:**

The control of foodborne pathogens, including *Escherichia coli* O157:H7, *Staphylococcus aureus*, *Salmonella* Typhimurium, *Pseudomonas aeruginosa*, and *Bacillus cereus*, represents a critical challenge in food safety, necessitating the development of novel antimicrobial agents with both efficacy and safety. In this study, hyperbranched poly-D-mannose was employed as a stabilizer to successfully synthesize silver nanoparticles with exceptional stability and dispersibility. The prepared PM-AgNPs demonstrated remarkable bactericidal and anti-biofilm activities while circumventing the use of potentially hazardous chemical stabilizers commonly employed in conventional nanoparticle synthesis. This advancement provides an innovative solution for pathogenic microorganism control during food processing and storage, offering a combination of high antimicrobial efficacy and biosafety. The excellent biocompatibility and controlled release characteristics of this system make it particularly suitable for preservation applications in high-risk food products such as meat and dairy items, thereby significantly contributing to the enhancement of food safety standards.

## INTRODUCTION

Food safety stands as a pivotal concern in global public health, with diseases stemming from it exerting profound impacts on human health and life (1). According to the World Health Organization, an alarming estimate of approximately 10% of the global population falls ill annually due to the consumption of contaminated food (2). *Escherichia coli*, *Staphylococcus aureus*, *Salmonella*, and *Listeria monocytogenes* are prevalent foodborne pathogens (3). These pathogens not only contaminate food but also produce harmful toxins, posing a significant threat to human health (4). To date, over 200 distinct foodborne illnesses have been identified, with the most severe manifestations typically affecting the elderly, children, immunocompromised individuals, and healthy persons exposed to high concentrations of these pathogens (5). Microbial contamination of food can occur at various stages throughout the “farm-to-table” continuum, including harvesting, slaughter, processing, and distribution, often stemming from environmental pollution sources such as water, soil, or air (6). Traditional preservation methods like salting, drying, freezing, or fermentation may help extend food shelf life, yet they do not fully mitigate the risk of subsequent contamination. Hence, the strategic use of antimicrobial agents during crucial stages of food processing and packaging has emerged as a vital measure to safeguard food safety (7).

At present, chemical antimicrobials such as sodium benzoate, potassium sorbate, and nitrites are applied in the food industry (8). Although they exhibit broad-spectrum bactericidal effects, excessive or inappropriate use of these chemical antimicrobials can potentially induce bacterial resistance, posing a health risk (9). In light of the adverse effects associated with synthetic antimicrobials and the growing crisis of antimicrobial resistance, there has been a notable shift toward exploring and utilizing compounds inherently possessing antimicrobial activities. Among the naturally occurring antimicrobials widely used today are polyphenolic compounds, volatile essential oils, enzymatic lysozymes, biologically active antimicrobial peptides, chitin-derived polysaccharide chitosan, and bacteriocins—ribosomally synthesized peptide antibiotics (10). However, these natural antimicrobials face practical challenges, as they are often sensitive to storage conditions and slight variations in production processes, which may diminish their effectiveness. Furthermore, studies suggest that certain natural antimicrobials may interact undesirably with specific food components, further impacting their application efficacy and food safety (11). In recent years, nanoparticles have emerged as a promising alternative to conventional antimicrobial substances, demonstrating significant potential in inhibiting or eradicating pathogenic bacteria (12). Nanostructures serve as excellent carriers, capable of shielding bioactive compounds from adverse conditions during food production and storage, thereby enhancing the stability and efficacy of these compounds against pathogenic bacteria. Additionally, nanostructures can regulate the release rate of antimicrobial compounds, ensuring that a sufficient amount of active ingredients is timely released from the nanostructures to penetrate and act upon the surface or interior regions of food matrices where microbial activities are prevalent, achieving effective inhibition or elimination of pathogenic bacteria (11).

Among the various nanomaterials, silver nanoparticles (AgNPs) exhibit remarkable antimicrobial properties, low toxicity, and exceptional biocompatibility, positioning them as a highly promising antimicrobial nanomaterial within the food industry (13). However, their inherently vast specific surface area makes AgNPs highly susceptible to instability and prone to aggregation, a phenomenon that can drastically impair their antimicrobial effectiveness. As such, ensuring their adequate dispersion in liquid systems is crucial. An efficient strategy involves incorporating stabilizers, which can effectively prevent AgNPs aggregation and manage their size. A wide array of stabilizers, including surfactants, sodium citrate, polysaccharides, and proteins, is commonly utilized in the synthesis of AgNPs (14–17). Notably, polysaccharides stand out as ideal green soft templates for nanoparticle synthesis due to their safety, non-toxic nature, intricate branched structures, and reactive hydroxyl groups. These polysaccharides serve as protective coatings on the nanoparticle surface, efficiently hindering aggregation by creating steric barriers and forming hydrogen bonds with the nanoparticles, thereby ensuring the stability and dispersion of the nanoparticles (18, 19). Additionally, numerous polysaccharides have been proven to possess exceptional biocompatibility, along with diverse biological activities such as antioxidant, immunomodulatory, and antibacterial properties. Therefore, the utilization of polysaccharides in the preparation of AgNPs has the potential to amplify the benefits of silver and elicit synergistic effects.

A diverse range of naturally derived polysaccharides, such as cellulose (20), chitosan (21), sodium alginate (22), and exopolysaccharides sourced from lactic acid bacteria (23), has proven effective as stabilizers or reducing agents in the synthesis process of silver nanoparticles, which predominantly exhibit linear or low-branched structural characteristics. The extended chain conformation of these polysaccharides occupies a large hydrodynamic volume in solution, restricting molecular chain mobility and resulting in high system viscosity. Simultaneously, the densely distributed hydroxyl groups along their molecular chains readily form intramolecular/intermolecular hydrogen-bonding networks. This strong self-association effect markedly diminishes their solvation capacity with aqueous media, leading to significantly limited solubility. Collectively, these structural characteristics render such polysaccharides ineffective in achieving ideal dispersion stability within silver nanoparticle synthesis systems. In contrast, hyperbranched polysaccharides represent a class of carbohydrate polymers featuring three-dimensional multibranched topological structures. Their molecular chains form hierarchical branching networks through ABx-type (*x* ≥ 2) asymmetric branching units (24). Compared to linear or sparsely branched polysaccharides, their highly branched architecture adopts compact globular conformations in solution, with dramatically reduced interchain entanglement, thereby exhibiting exceptional aqueous solubility and dispersion stability. These unique carbohydrate polymers spontaneously assemble into distinctive “micelle-like structures” through chemical bonding, comprising hydrophobic cores and hydrophilic shells. This structural motif creates ideal cavities for encapsulating silver nanoparticles, enabling superior stabilization (25).

In our previous study, D-mannose served as the monomeric building block for synthesizing a substantial quantity of non-toxic, multibranched poly-D-mannose (PM) via solid-phase polycondensation. This synthesized poly-D-mannose exhibited inhibitory activity against biofilms formed by common pathogenic bacteria (26). Building upon this groundwork, the present study utilized poly-D-mannose as a stabilizer, along with silver nitrate as the silver source and sodium borohydride as the reductant, to synthesize PM-AgNPs. Structural analysis of these synthesized poly-D-mannose-silver nanoparticles was then conducted to elucidate the mechanism through which poly-D-mannose stabilizes the silver nanoparticles. Furthermore, this study selected prevalent foodborne pathogenic bacteria to assess the antibacterial and antibiofilm properties of the poly-D-mannose-silver nanoparticles. The objective of this research is to provide data support for the application of poly-D-mannose as a stabilizer in the synthesis of silver nanoparticles and the potential use of poly-D-mannose-silver nanoparticles as antibacterial materials in the food industry.

## MATERIALS AND METHODS

### Materials

*Bacillus cereus* (CICC 21261), *Escherichia coli* O157:H7 (CICC 10907), and *Salmonella* Typhimurium (CICC 22956/ATCC 14028) were purchased from China Center of Industrial Culture Collection (CICC). *Staphylococcus aureus* (CGMCC 1.291) was purchased from China General Microbiological Culture Collection Center (CGMCC). *Pseudomonas aeruginosa* (ATCC 27853) was purchased from American Type Culture Collection (ATCC). All chemical reagents used were analytical grade.

Poly-D-mannose was prepared by our own laboratory (26).

### Preparation of PM-AgNPs

Three milliliters of PM solutions with different concentrations (0.5–40 mg/mL) was mixed with 10 mL, 20 mM silver nitrate and stirred for 10 min on a heated magnetic stirrer at 70°C, 600 rpm. The sodium borohydride solution was slowly added dropwise to the above mixture at a molar ratio of 2:1 with silver nitrate, and the reaction was allowed to proceed for 1 h. The reaction solution without PM addition served as the control. After the reaction, the solution was dialyzed in a 500 Da (Biotopped, Beijing, China) dialysis bag for 3–4 days to obtain PM-AgNPs.

### Structural characterization of PM-AgNPs

#### Ultraviolet-visible absorption spectrometry of PM-AgNPs

UV-visible spectroscopy is a highly significant and straightforward technique for confirming the formation of nanoparticles. Silver nanoparticles exhibit a strong absorption band in solution due to surface plasmon resonance, resulting in the specific color. One hundred microliters of PM-AgNPs solution was dispensed into a 96-well plate. Subsequently, the absorption spectrum of the PM-AgNPs within the wavelength range of 200–700 nm was measured using a microplate reader (Epoch 2, BioTek, USA).

#### Particle size distribution and zeta potential of PM-AgNPs

The size and zeta potential of PM-AgNPs were measured using the Nano ZS zetasizer system (Malvern, UK). The specific measurement conditions employed are as follows: a particle size measurement angle of 173°, a measurement temperature of 25°C, a solvent viscosity of 0.8872 mPa s, a solvent refractive index of 1.330, and a material refractive index of 1.59. For the measurement, 1 mL of PM-AgNPs was placed in the sample cell. Each sample was measured three times consecutively to ensure the accuracy of the results.

#### Observation of the morphology of PM-AgNPs by transmission electron microscopy

PM-AgNPs were dripped onto a copper grid with a carbon support film, dried at room temperature, and then the size and morphology of PM-AgNPs were observed using the transmission electron microscopy (TEM) (Tecnai G2 20 S-TWIN, FEI, USA).

#### Fourier transform infrared spectroscopy of PM-AgNPs

The potassium bromide pellet pressing technique was employed for conducting Fourier transform infrared (FT-IR) spectroscopy measurements of PM-AgNPs using a Vertex 70V instrument equipped with a Hyperion 2000 (Bruker, Germany).

#### Elemental analysis of PM-AgNPs using energy-dispersive X-ray spectroscopy

After freeze-drying the PM-AgNPs solution, the surface elemental composition of PM-AgNPs was analyzed using an energy-dispersive X-ray spectroscopy (EDX) device (X-MaxN50 Aztec, JEOL, Japan).

### Inhibitory activity of PM-AgNPs against pathogenic bacteria

Five common pathogens, including *E. coli* O157:H7, *S.* Typhimurium, *P. aeruginosa*, *S. aureus*, and *B. cereus*, were inoculated into Luria-Bertani (LB) liquid medium at a concentration of 1% and subsequently activated twice at 37°C with shaking. Following activation, they were transferred into a 96-well plate (Costar, Corning, USA) for further processing. Different concentrations of PM-AgNPs solutions prepared with sterile water (final concentrations: 11.9–380 µg/mL) were added to the 96-well plate containing the aforementioned pathogens. A negative control group was established by adding only sterile water, while a blank control group consisted of LB medium containing the sample but devoid of any pathogens. Each sample was tested in triplicate to ensure accuracy. The 96-well plate containing varying concentrations of PM-AgNPs was incubated in a microplate reader under continuous shaking for 24 h to obtain the growth kinetics curves of the pathogens.

### Inhibitory activity of PM-AgNPs against pathogenic bacterial biofilm

The effect of PM-AgNPs on the biofilm formation of *E. coli* O157:H7, *S.* Typhimurium, *P. aeruginosa*, *S. aureus*, and *B. cereus* was determined at concentrations below MICs. These five pathogens were inoculated into tryptic soy broth liquid medium containing 1.7% tryptone, 0.3% soya peptone, 0.25% K_2_HPO_4_, 0.25% glucose, and 0.5% NaCl and incubated with agitation at 37°C. Following two rounds of revitalization, the bacterial suspension was adjusted to an optical density (OD) of 0.2 and then diluted 10-fold. Next, 90 µL (approximately 10^6^ CFU/mL) of this suspension was dispensed into each well of a 96-well plate (Costar, Corning, USA), and 10 µL of PM-AgNPs dissolved in sterile water at different concentrations was added to each well. Each treatment condition was replicated in quadruplicate. Bacterial suspensions treated with sterile water were set as the negative control, whereas medium containing the sample but no bacterial suspension was used as the blank control. The 96-well plate was then incubated statically at 37°C for 24 h. After incubation, the culture supernatant was aspirated from each well, and these wells were thoroughly washed four times with 120 µL of sterile phosphate-buffered saline to remove non-adherent bacteria. Subsequently, 100 µL of the 0.5 mg/mL solution of 3-(4,5-dimethylthiazol-2-yl)-2,5-diphenyltetrazolium bromide (MTT) was added to each well to stain the bacteria adhering to the bottom and walls of the wells. The plate was incubated in the dark at 37°C for 3 h. Following incubation, the MTT solution was discarded, and 100 µL of dimethyl sulfoxide was added to each well. The plate was gently agitated for 10 min to dissolve the formazan crystals formed by viable bacteria. The absorbance of each well was measured at a wavelength of 490 nm using a microplate reader (Epoch 2, BioTek, USA). The biofilm formation rate of the negative control was designated as 100%, and the biofilm formation rate under various concentrations of PM-AgNPs was calculated using the following formula: Biofilm formation rate = (OD_sample_/OD_negative_) × 100%.

### Statistical analysis

Data were processed using Origin 2021. The experiment was repeated three times, and the experimental results were expressed as mean ± standard deviation.

## RESULTS

### Synthesis of PM-AgNPs

**Fig 1:**
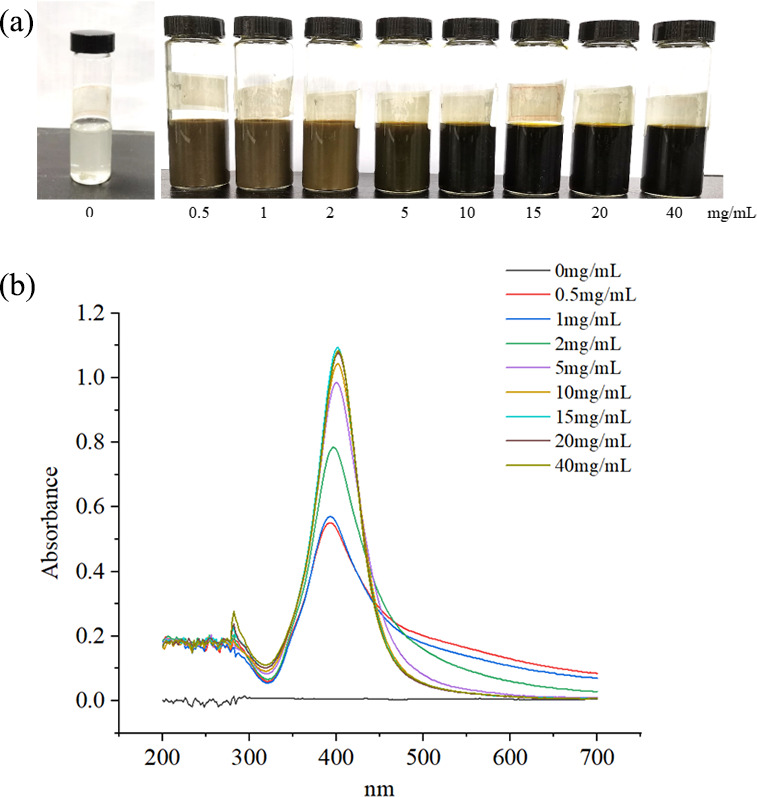
(**a**) AgNPs stabilized by different concentrations of poly-D-mannose and (**b**) UV-Vis spectra of AgNPs stabilized with different concentrations of poly-D-mannose.

Silver nanoparticles stabilized by poly-D-mannose were synthesized across various concentrations, as illustrated in Fig. 1a, and their presence was confirmed through UV-Vis spectroscopy, as depicted in Fig. 1b. In Fig. 1a, when PM was absent, rapid agglomeration and sedimentation of AgNPs were observed. However, upon incorporating PM into the reaction mixture, the AgNPs became dispersed. Within a PM concentration range of 0.5–2 mg/mL, the PM-AgNPs existed in a colloidal suspension state, rendering the solution opaque. As the concentration climbed to 5 mg/mL, the PM-AgNPs solution displayed enhanced translucency. Further incrementing the concentration to 10–40 mg/mL resulted in a dark red, translucent PM-AgNPs solution, suggesting changes in particle dispersion and optical properties. In Fig. 1b, within the spectral range of 200–700 nm, no absorption was noted in the absence of PM, indicating the absence of synthesized AgNPs. Upon adding PM, an absorption peak emerged near 400 nm. At PM concentrations of 0.5 mg/mL and 1 mg/mL, the absorption peak exhibited a broad distribution with relatively low peak heights, suggesting the presence of AgNPs but with larger particle sizes and an uneven distribution. As the PM concentration increased, the absorption peak narrowed and its peak height increased. Beyond 10 mg/mL, any subsequent increase in PM concentration had minimal impact on the peak profile.

### Characterization of PM-AgNPs

**Fig 2:**
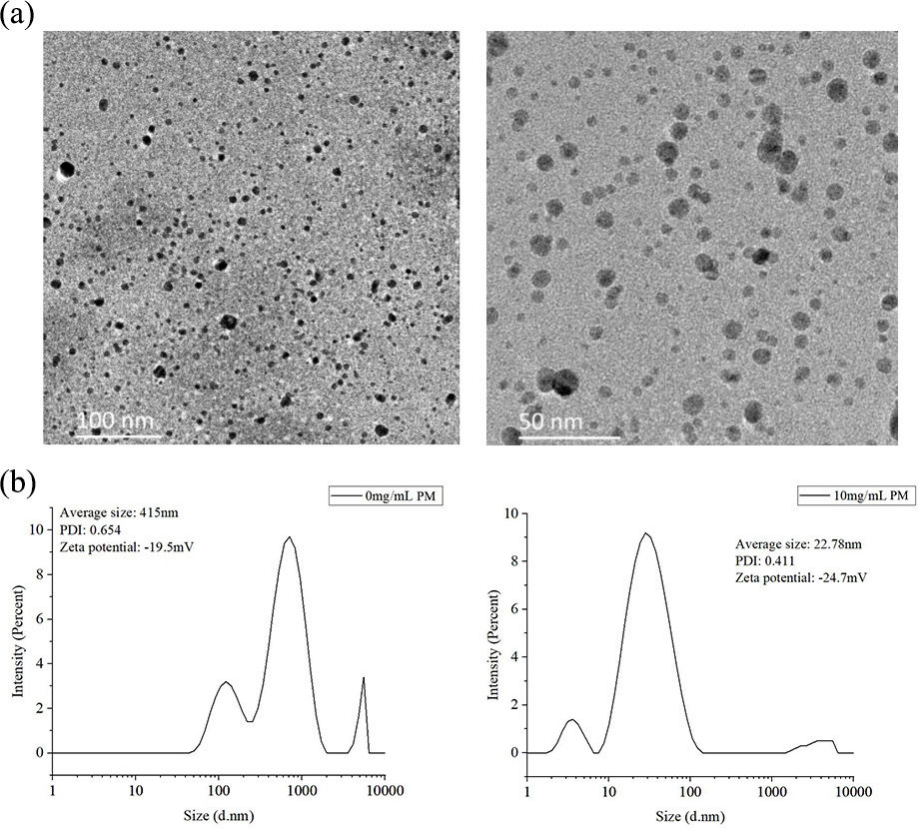
(**a**) TEM image of AgNPs stabilized with 10 mg/mL poly-D-mannose. (**b**) Particle size, polydispersity index (PDI), and zeta potential of AgNPs synthesized without and with 10 mg/mL poly-D-mannose.

To investigate the morphology and particle size of PM-AgNPs, we conducted additional analyses using the TEM and dynamic light scattering (DLS) analyzer. The TEM image in Fig. 2a revealed that PM-AgNPs exhibited an irregular spherical morphology, with particle sizes ranging from 2 to 18 nm. The DLS analysis presented in Fig. 2b demonstrated that the PM-AgNPs had an average particle diameter of 22.78 nm and a polydispersity index of 0.411, along with a zeta potential of −24.7 mV.

**Fig 3:**
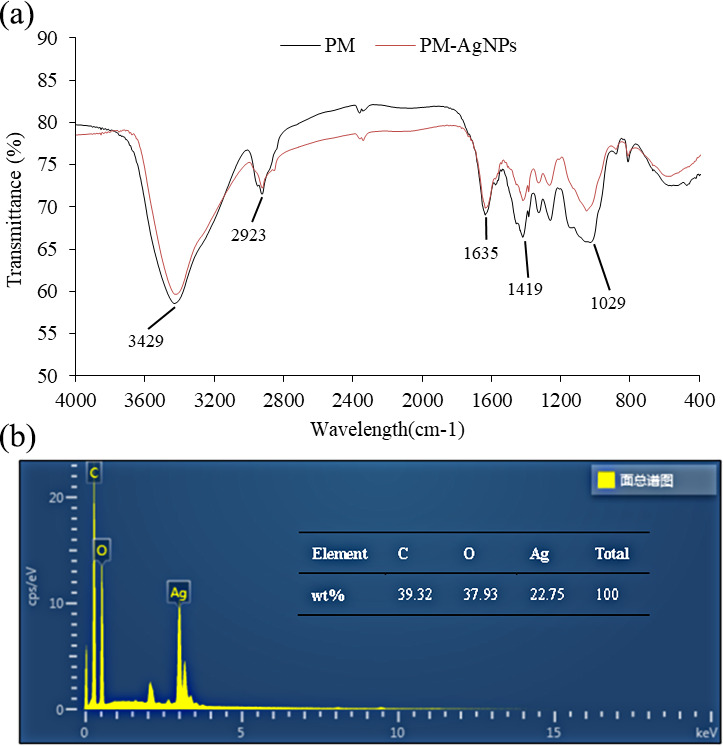
(**a**) FT-IR of poly-D-mannose (PM) and silver nanoparticles stabilized with 10 mg/mL poly-D-mannose (PM-AgNPs). (**b**) EDX analysis of AgNPs stabilized with 10 mg/mL poly-D-mannose.

FT-IR spectroscopy was used to further explore the changes in chemical structure and possible roles of PM in the synthesis of AgNPs. The FT-IR spectra of PM and PM-AgNPs are shown in Fig. 3a. The FT-IR spectrum of PM displayed a strong absorption band at 3,429 cm⁻¹, characteristic of O–H stretching vibration (27). The absorption peak at 2,923 cm⁻¹ corresponded to the asymmetric stretching vibration of C–H bonds (28). The peak at 1,635 cm⁻¹ was associated with the asymmetric stretching vibration of C⚌O, while the peak at 1,419 cm⁻¹ represented the symmetric stretching vibration of C⚌O (29, 30). The absorption peak at 1,029 cm⁻¹ arose from the combined effect of C–O stretching vibration and C–O–C vibration (31). In the FT-IR spectrum of PM-AgNPs, the bands at 2,923 cm⁻¹, 1,635 cm⁻¹, and 1,419 cm⁻¹ remained unchanged despite the presence of AgNPs. However, the formation of AgNPs within the PM matrix led to a slight red shift in the position at 3,429 cm⁻¹, which may have been attributed to interactions between the nanoparticles and the O–H groups in the PM structure (32). Furthermore, the absorption peak of PM originally observed at 1,029 cm⁻¹ underwent a blue shift to 1,051 cm⁻¹ in the presence of AgNPs, suggesting a strong interaction between the AgNPs and the C–O or C–O–C bonds within the PM structure (33).

The elemental composition on the surface of PM-AgNPs was determined by EDX, as shown in Fig. 3b. The elemental ratio in PM-AgNPs was found to be C:O:Ag = 39.32:37.93:22.75.

### Anti-bacterial activities of PM-AgNPs

**Fig 4:**
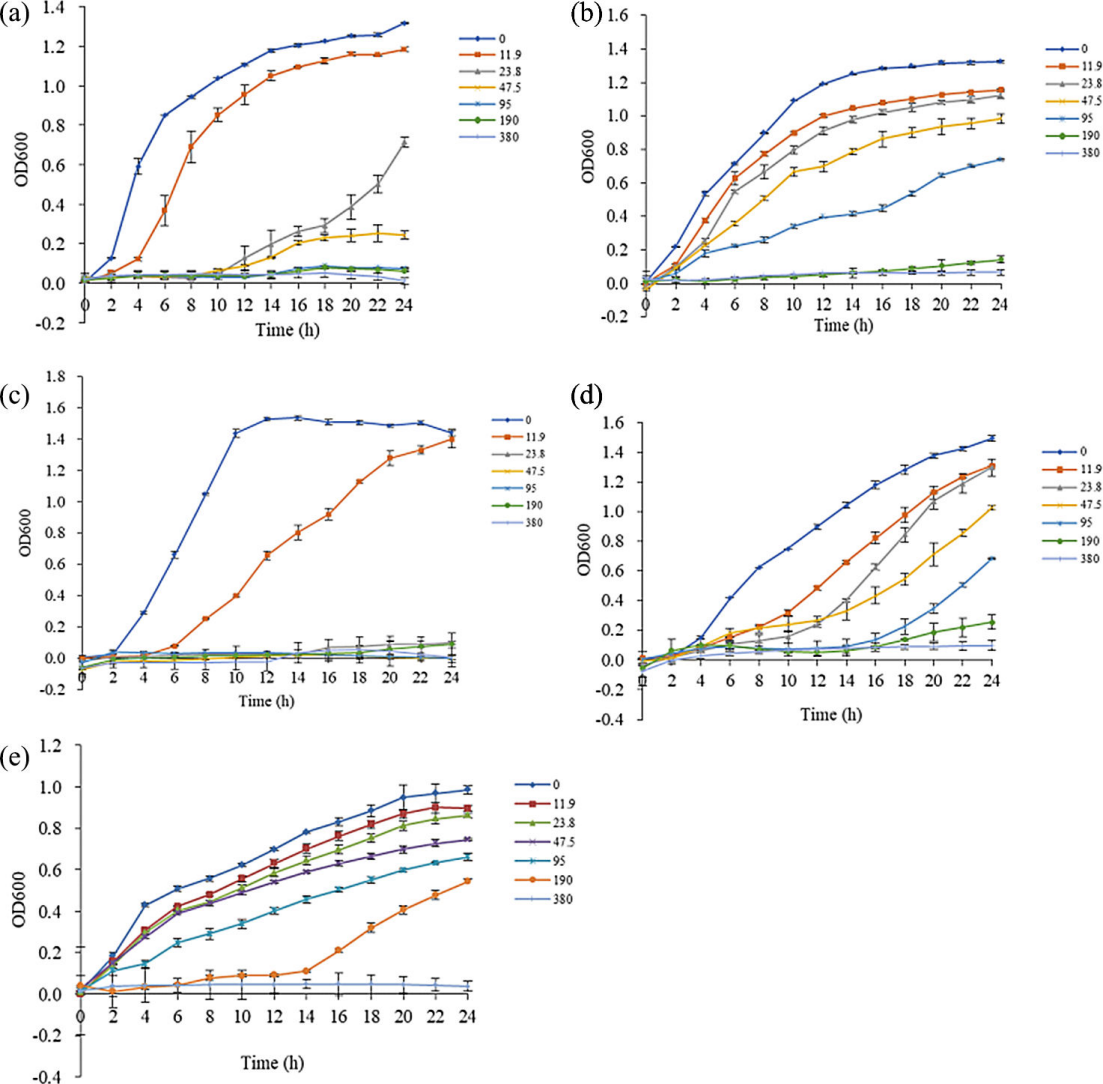
Effect of different concentrations of PM-AgNPs on pathogen growth. (**a**) *E. coli* O157:H7, (**b**) *S.* Typhimurium, (**c**) *P. aeruginosa*, (**d**) *S. aureus*, and (**e**) *B. cereus*.

In this study, the effects of PM-AgNPs on the growth of five common foodborne pathogens, including *E. coli* O157:H7, *S.* Typhimurium, *P. aeruginosa*, *S. aureus*, and *B. cereus*, were evaluated. The results are shown in Fig. 4. Compared to the control group without PM-AgNPs, the presence of PM-AgNPs significantly affected the cellular viability of all pathogenic strains. As the concentration of PM-AgNPs escalated, a concentration-dependent decline in cellular viability was evident. The ranking of PM-AgNPs' inhibitory effect on these five pathogenic bacteria was as follows: *P. aeruginosa* exhibited the greatest susceptibility, followed by *E. coli* O157:H7, *S.* Typhimurium, *S. aureus*, and *B. cereus*, in descending order. The MICs of PM-AgNPs against these pathogens were as follows:

- *P. aeruginosa*: 23.8 µg/mL
- *E. coli* O157:H7: 95 µg/mL
- *S.* Typhimurium: 190 µg/mL
- *S. aureus*: 380 µg/mL
- *B. cereus*: 380 µg/mL

### Anti-biofilm activities of PM-AgNPs

**Fig 5:**
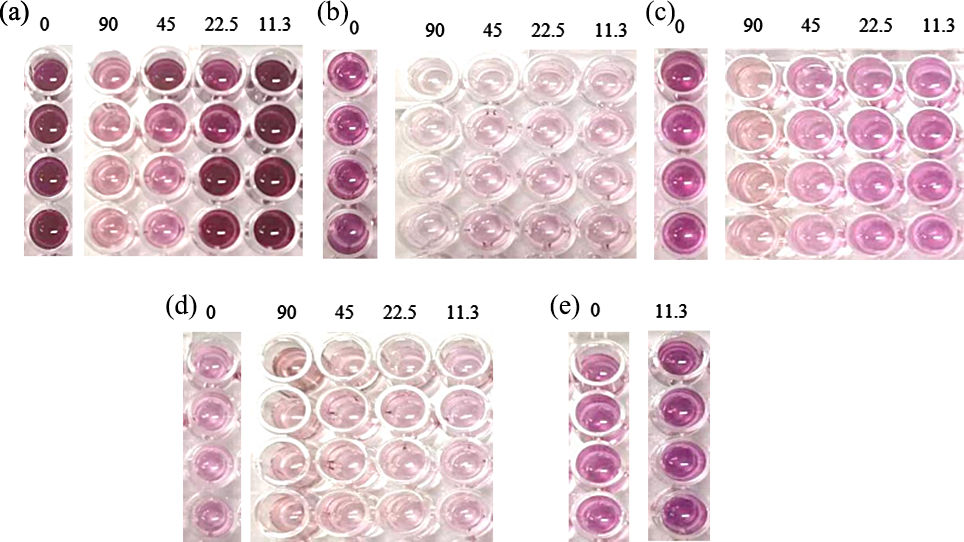
Changes in color of the pathogen biofilm after MTT staining with different concentrations of PM-AgNPs. (**a**) *B. cereus*, (**b**) *S. aureus*, (**c**) *S.* Typhimurium, (**d**) *E. coli* O157:H7, and (**e**) *P. aeruginosa*.

Utilizing the microtiter plate assay, the influence of PM-AgNPs on the biofilm formation capabilities of the five pathogenic bacteria was assessed at concentrations below their MICs. The results are exhibited in Fig. 5 and Table 1. Based on color observation and data analysis, PM-AgNPs demonstrated the strongest inhibitory effect on *S. aureus* biofilm formation, with a biofilm formation rate of just 1.01% at a concentration of 11.3 µg/mL, suggesting nearly complete suppression of biofilm development. Within the concentration range of 11.3–90 µg/mL, no significant variations in the biofilm formation were observed for *S. aureus. S.* Typhimurium exhibited the second highest sensitivity, with a dose-dependent reduction in the biofilm formation as the PM-AgNPs concentration increased, reaching a biofilm formation rate of 17.84% at the highest concentration of 90 µg/mL. The effect of PM-AgNPs on *E. coli* O157:H7 biofilm formation was not concentration-dependent, with the lowest biofilm formation rate of 38.29% recorded across the concentration range of 11.3–90 µg/mL. At lower concentrations of PM-AgNPs, *B. cereus* biofilm formation exceeded that of the negative control, indicating an enhancement. However, as the concentration of PM-AgNPs increased, biofilm formation gradually decreased, reaching a formation rate of 24.69% at the concentration of 90 µg/mL. It is noteworthy that PM-AgNPs failed to inhibit *P. aeruginosa* biofilm formation; instead, at the concentration of 11.3 µg/mL, it promoted biofilm development.

**TABLE 1:** Effects of different concentrations of PM-AgNPs on the pathogen biofilm

PM-AgNPs (µg/mL)	Amount of biofilm formation (%) by:				
	*B. cereus*	*S. aureus*	*S.* Typhimurium	*E. coli* O157:H7	*P. aeruginosa*
11.3	119.67% ± 16.05%	1.01% ± 0.06%	48.03% ± 15.67%	47.75% ± 9.02%	151.6% ± 14.3%
22.5	117.93% ± 19.29%	2.42% ± 1.92%	44.94% ± 11.32%	38.29% ± 9.52%	
45	68.93% ± 21.56%	1.40% ± 1.11%	30.70% ± 11.13%	41.97% ± 10.79%	
90	24.69% ± 7.34%	1.17% ± 1.20%	17.84% ± 3.59%	40.89% ± 1.04%	

## DISCUSSION

Poly-D-mannose is a hyperbranched polysaccharide featuring a multibranched structure with a high degree of branching up to 0.53, and it comprises 11 distinct types of saccharide residues (26). This unique polysaccharide structure endows poly-D-mannose with exceptional water-solubility properties, abundant spatial cavities, numerous terminal hydroxyl functional groups, and a high specific surface area, collectively providing an ideal microenvironment for the growth of silver nanoparticles. The hyperbranched architecture of poly-D-mannose confers exceptional water solubility, offering distinct advantages over reported polysaccharide-based AgNPs stabilizing systems, such as chitosan, alginate, and starch, in the preparation of AgNPs. Chitosan forms a compact, dense structure due to strong intramolecular and intermolecular hydrogen bonding, rendering it soluble only in acidic media (34). This property not only restricts its use in neutral or alkaline reaction environments but may also induce localized pH-sensitive aggregation during AgNPs synthesis. The high viscosity and pronounced swelling behavior of alginate hinder ion mass transfer and diffusion during the nucleation and growth stages of AgNPs, leading to increased particle size polydispersity (35, 36). Meanwhile, the poor solubility and dispersibility of native starch often result in an unstable colloidal system, prone to uncontrolled aggregation of AgNPs, thereby compromising their long-term stability and functionality (37, 38). Conversely, the inherent hyperbranched structure and abundant exposed hydroxyl groups of poly-D-mannose enable efficient dissolution and molecular-level dispersion across a broad pH range. This capability creates a uniform and stable microenvironment for silver nanoparticles, greatly improving both the controllability of the AgNPs preparation process and the quality of the resulting product. As revealed by the FT-IR results, silver nanoparticles were stabilized by poly-D-mannose primarily through the formation of an interaction network comprising O–H⋯Ag hydrogen bonds and C–O⋯Ag coordination bonds, effectively inhibiting their aggregation. This stabilization mechanism differs from those of other common polysaccharides. In starch, the majority of hydroxyl groups are embedded within linear chains or helical structures, resulting in limited accessible active sites for silver coordination. Its stabilization primarily relies on hydrogen bond reorganization to form an encapsulated “core-shell” structure (39), which exhibits relatively poor stability under changing environmental conditions and is prone to aggregation. Alginate mainly depends on electrostatic interactions through deprotonated carboxyl groups to stabilize silver nanoparticles, a process that requires an alkaline environment (40). Chitosan achieves stabilization primarily via coordination between its amino groups and silver (41). In contrast, poly-D-mannose, leveraging its hyperbranched architecture, provides a multi-anchoring, networked binding mode that offers more robust and durable protection for silver nanoparticles.

The concentration of poly-D-mannose played a pivotal role in the formation of silver nanoparticles. As the quantity of poly-D-mannose was increased, a higher number of hydroxyl groups were adsorbed onto the surfaces of nascent silver nuclei. The resulting spatial hindrance effectively shielded the formed Ag nanoparticles, thereby hindering their aggregation. Simultaneously, poly-D-mannose encapsulated the surfaces of Ag nanoparticles promptly when their particle sizes were very small, protecting the formed Ag nanoparticles and reducing their particle sizes. Therefore, the addition of poly-D-mannose significantly enhanced the dispersion of silver nanoparticles and refined the particles to a certain extent. When the concentration of poly-D-mannose surpassed 10 mg/mL, the change in the UV-Vis absorption peak became less notable, suggesting that when the quantity of poly-D-mannose reached a certain threshold, the hydroxyl groups in poly-D-mannose were just sufficient to form a protective layer on the surfaces of all the silver nuclei produced during the reaction. After this point, further addition of poly-D-mannose did not exert additional inhibitory effects on particle enlargement. These results are consistent with several previous reports for the fabrication of AgNPs with polysaccharides as reducing and/or stabilizing agents, such as starch, chitosan, soluble soybean polysaccharide, etc. (42–44). In this study, the average particle size of AgNPs determined by DLS was 22 nm, which was larger than the average particle size of 2–18 nm measured by TEM. The difference primarily arises because DLS is a highly sensitive technique that quantifies both large and small particles, as well as aggregates present in a solution, while TEM exclusively measures individual particles, potentially leading to biases during the sample preparation stage (45). Additionally, DLS calculates the hydrodynamic diameter of NPs, encompassing the core and any attached particles on their surfaces (46). Furthermore, the interaction between particles and the stabilizing agent may also have an impact on the hydrodynamic diameter (47).

PM-AgNPs exhibited inhibitory effects on selected common gram-positive and gram-negative bacteria. Beyond its exceptional stabilizing capacity, the poly-D-mannose may also play an active role in enhancing the antibacterial efficacy of AgNPs. The antibacterial mechanisms of AgNPs, such as penetration of cell membranes, induction of reactive oxygen species (ROS) resulting in oxidative stress, and interference with microbial signal transduction, have been extensively studied (48). It is particularly noteworthy that the introduction of poly-D-mannose not only stabilizes AgNPs but may also enhance antibacterial activity through a unique synergistic mechanism. As a mannose-rich polysaccharide, poly-D-mannose itself acts as a target for multiple bacterial adhesins and possesses intrinsic anti-adhesion potential (49). When coated on the surface of AgNPs, it enables targeted binding to mannose-specific lectins on bacterial surfaces, achieving more efficient membrane association and directional delivery of Ag^+^. This active targeting capability is absent in non-specific polysaccharide stabilizers such as starch, chitosan, and alginate. Our previous research has further confirmed that poly-D-mannose effectively inhibits pathogenic biofilm formation by suppressing bacterial adhesion (50). Based on this foundation, we propose that PM-AgNPs exert antibacterial effects via the following synergistic pathway: firstly, the mannose units on the surface of poly-D-mannose facilitate specific multivalent binding to bacterial lectins, enabling active targeting and accumulation on pathogens; poly-D-mannose competitively inhibits bacterial adhesion, disrupts quorum sensing systems, and penetrates biofilms to disrupt their matrix structure. Subsequently, within the microenvironment preconditioned by poly-D-mannose, the silver nanoparticles release Ag^+^ more efficiently and catalyze the generation of substantial ROS, thereby synergistically inducing multiple lethal effects, including cell membrane rupture, inactivation of intracellular biomacromolecules, and oxidative damage. Thus, the “active targeting and biofilm-inhibiting” capabilities of poly-D-mannose and the “multimodal antimicrobial” mechanisms of AgNPs exhibit spatiotemporal synergy, collectively mediating highly efficient eradication of pathogenic bacteria.

Furthermore, we found that gram-negative bacteria displayed greater sensitivity to PM-AgNPs compared to gram-positive bacteria. This difference may be attributed to variations in membrane structure and cell wall composition, which in turn affect the entry of AgNPs (51, 52). Because of the structural differences in the cell walls between gram-negative and gram-positive bacteria, with the cell wall of gram-negative bacteria being more fragile, AgNPs are more readily able to penetrate their cell walls and establish direct contact with the cell membrane, thereby exerting a more effective bacteriostatic effect (53). On the other hand, PM-AgNPs may release silver cations, which exhibit a propensity to adsorb onto the negatively charged cell walls of gram-negative bacteria. This adsorption process further induces deformation and disruption of the cell membrane, leading to uncontrolled transport processes across the cytoplasmic membrane and ultimately causing complete bacterial death (54, 55). The inhibitory effects of PM-AgNPs on three types of gram-negative bacteria also exhibited certain differences, which are likely attributed to the inherent variations among different bacteria. These variations result in varying sensitivities to PM-AgNPs, thereby influencing the antibacterial efficacy of PM-AgNPs.

At concentrations below the MICs, PM-AgNPs displayed varying degrees of inhibition on the biofilms of five pathogenic bacteria. The research by Mohanty et al. revealed that starch-stabilized nanoparticles (approximately 20 nm in diameter) effectively decreased biofilm formation by *P. aeruginosa* and *S. aureus* by 65% and 88%, respectively, at very low concentrations ranging from 1 to 2 mM (56). Moreover, Patel et al. found that chitosan-gel-stabilized silver nanoparticles, with an average particle size of 15 nm, exhibited dose-dependent inhibitory activity against the biofilms of *E. coli* and *S. aureus* within a concentration range of 10–100 µg/mL (57). The particle size of the AgNPs reported in these literatures is consistent with the PM-AgNPs prepared in our study, and they also exhibit similar inhibitory effects on the biofilms of pathogenic bacteria. Numerous studies have reported that AgNPs inhibit the formation of biofilms by disrupting their key components, which primarily include extracellular DNA (eDNA), extracellular proteins, and extracellular polysaccharides. Jiang and Ran reported that AgNPs can interact with the eDNA structures within biofilms through electrostatic, hydrophobic, and van der Waals interactions, thereby suppressing biofilm formation (58). Furthermore, AgNPs engage in interactions with a variety of proteins involved in quorum sensing, amyloidogenesis, metabolic processes, and membrane protein formation, through electrostatic, hydrophobic, hydrogen bonding, van der Waals, and π-π interactions. These interactions can lead to the inactivation or alteration of protein functions, further influencing biofilm formation (59–63). There are varying experimental results regarding the effect of nanoparticles on extracellular polysaccharides in biofilms. Dunsing et al. observed that the biofilm of the plant pathogen *Pantoea stewartii* interacted with nanoparticles, hindering their penetration into the biofilm matrix (64). In contrast, Kalishwaralal et al. found that AgNPs reduced the synthesis of extracellular polymeric substances (EPS) in *P. aeruginosa* and *S. epidermidis* biofilms through unknown mechanisms (65). Based on the established mechanisms underlying AgNPs-mediated biofilm inhibition and the unique structural characteristics of poly-D-mannose, we hypothesize that the anti-biofilm efficacy of PM-AgNPs may originate from a synergistic mechanism that potentiates these known pathways. The three-dimensional, highly branched architecture and abundant terminal hydroxyl groups of poly-D-mannose facilitate multivalent binding with EPS, thereby compromising the integrity of the biofilm matrix and enhancing the deep penetration capacity of the nanoparticles. Concurrently, surface-exposed mannose residues enable specific recognition and binding to lectin proteins on pathogenic bacteria, which not only promotes targeted accumulation of PM-AgNPs within the biofilm but may also interfere with quorum sensing systems and biofilm-associated gene expression. This combination of enhanced physical penetration and biological targeting distinguishes PM-AgNPs from systems employing conventional linear polysaccharides and offers a compelling explanation for their efficient biofilm suppression. Nevertheless, the specific contribution of each mechanism and their potential interdependencies requires further elucidation.

### Conclusion

In this study, poly-D-mannose was successfully employed as a stabilizer to prepare silver nanoparticles (PM-AgNPs) with a small particle size and good dispersion. These PM-AgNPs exhibited potent inhibitory activity against foodborne pathogenic bacteria and their biofilms, positioning them as promising antimicrobial agents for applications in food processing and preservation of food packaging materials. However, it is noteworthy that the specific antimicrobial and anti-biofilm mechanisms of PM-AgNPs have not been elucidated in this research. Therefore, subsequent studies should aim to further clarify the antimicrobial and anti-biofilm mechanisms of PM-AgNPs in order to provide a more solid theoretical foundation for their practical applications. Although PM-AgNPs demonstrate excellent *in vitro* activity, their performance in real food matrices, long-term stability, potential impact on sensory properties, and compliance with food safety regulations require further investigation before they can be deployed in practical settings.

## References

[1] Wei X, Zhao X. 2021. Advances in typing and identification of foodborne pathogens. Curr Opin Food Sci 37:52–57. doi:10.1016/j.cofs.2020.09.002

[2] Franz C, den Besten HMW, Böhnlein C, Gareis M, Zwietering MH, Fusco V. 2018. Microbial food safety in the 21st century: emerging challenges and foodborne pathogenic bacteria. Trends Food Sci Technol 81:155–158. doi:10.1016/j.tifs.2018.09.019

[3] Dai G, Yao H, Yang L, Ding Y, Du S, Shen H, Mo F. 2023. Rapid detection of foodborne pathogens in diverse foodstuffs by universal electrochemical aptasensor based on UiO-66 and methylene blue composites. Food Chem 424:136244. doi:10.1016/j.foodchem.2023.136244 37244183

[4] Imre K, Herman V, Morar A. 2020. Scientific achievements in the study of the occurrence and antimicrobial susceptibility profile of major foodborne pathogenic bacteria in foods and food processing environments in Romania: review of the last decade. Biomed Res Int 2020:5134764. doi:10.1155/2020/5134764 32685497 PMC7333035

[5] Bintsis T. 2017. Foodborne pathogens. AIMS Microbiol 3:529–563. doi:10.3934/microbiol.2017.3.529 31294175 PMC6604998

[6] Veskovic S. 2025. Natural food preservation: controlling loss, advancing safety

[7] Malhotra B, Keshwani A, Kharkwal H. 2015. Antimicrobial food packaging: potential and pitfalls. Front Microbiol 6:611. doi:10.3389/fmicb.2015.00611 26136740 PMC4468856

[8] Taylor TM, Davidson PM, David JR. 2020. Food antimicrobials–an introduction, p 1–12. In Antimicrobials in food. CRC Press.

[9] de Almeida Roger J, Magro M, Spagnolo S, Bonaiuto E, Baratella D, Fasolato L, Vianello F. 2018. Antimicrobial and magnetically removable tannic acid nanocarrier: a processing aid for Listeria monocytogenes treatment for food industry applications. Food Chem 267:430–436. doi:10.1016/j.foodchem.2017.06.109 29934188

[10] Pisoschi AM, Pop A, Georgescu C, Turcuş V, Olah NK, Mathe E. 2018. An overview of natural antimicrobials role in food. Eur J Med Chem 143:922–935. doi:10.1016/j.ejmech.2017.11.095 29227932

[11] Lopes NA, Brandelli A. 2018. Nanostructures for delivery of natural antimicrobials in food. Crit Rev Food Sci Nutr 58:2202–2212. doi:10.1080/10408398.2017.1308915 28394691

[12] López de Dicastillo C, Patiño Vidal C, Falcó I, Sánchez G, Márquez P, Escrig J. 2020. Antimicrobial bilayer nanocomposites based on the incorporation of as-synthetized hollow zinc oxide nanotubes. Nanomaterials (Basel) 10:503. doi:10.3390/nano10030503 32168893 PMC7153247

[13] He Y, Li H, Fei X, Peng L. 2021. Carboxymethyl cellulose/cellulose nanocrystals immobilized silver nanoparticles as an effective coating to improve barrier and antibacterial properties of paper for food packaging applications. Carbohydr Polym 252:117156. doi:10.1016/j.carbpol.2020.117156 33183607

[14] Lima BGA, Silva RR, Meira HM, Durval IJB, Macedo Bezerra Filho C, Silva TAL, Sarubbo LA, Luna JM. 2024. Synthesis and characterization of silver nanoparticles stabilized with biosurfactant and application as an antimicrobial agent. Microorganisms 12:1849. doi:10.3390/microorganisms12091849 39338522 PMC11433786

[15] Oprica L, Andries M, Sacarescu L, Popescu L, Pricop D, Creanga D, Balasoiu M. 2020. Citrate-silver nanoparticles and their impact on some environmental beneficial fungi. Saudi J Biol Sci 27:3365–3375. doi:10.1016/j.sjbs.2020.09.004 33304144 PMC7715440

[16] Hasanin M, Elbahnasawy MA, Shehabeldine AM, Hashem AH. 2021. Ecofriendly preparation of silver nanoparticles-based nanocomposite stabilized by polysaccharides with antibacterial, antifungal and antiviral activities. Biometals 34:1313–1328. doi:10.1007/s10534-021-00344-7 34564808 PMC8475443

[17] Zeng A, Wang B, Zhang C, Yang R, Yu S, Zhao W. 2022. Physicochemical properties and antibacterial application of silver nanoparticles stabilized by whey protein isolate. Food Biosci 46:101569. doi:10.1016/j.fbio.2022.101569

[18] Liu H, Zhang M, Meng F, Su C, Li J. 2023. Polysaccharide-based gold nanomaterials: synthesis mechanism, polysaccharide structure-effect, and anticancer activity. Carbohydr Polym 321:121284. doi:10.1016/j.carbpol.2023.121284 37739497

[19] Madani M, Hosny S, Alshangiti DM, Nady N, Alkhursani SA, Alkhaldi H, Al-Gahtany SA, Ghobashy MM, Gaber GA. 2022. Green synthesis of nanoparticles for varied applications: green renewable resources and energy-efficient synthetic routes. Nanotechnol Rev 11:731–759. doi:10.1515/ntrev-2022-0034

[20] Zeng A, Yang R, Tong Y, Zhao W. 2023. Functional bacterial cellulose nanofibrils with silver nanoparticles and its antibacterial application. Int J Biol Macromol 235:123739. doi:10.1016/j.ijbiomac.2023.123739 36806768

[21] Affes S, Maalej H, Aranaz I, Kchaou H, Acosta N, Heras Á, Nasri M. 2020. Controlled size green synthesis of bioactive silver nanoparticles assisted by chitosan and its derivatives and their application in biofilm preparation. Carbohydr Polym 236:116063. doi:10.1016/j.carbpol.2020.116063 32172878

[22] Tian S, Hu Y, Chen X, Liu C, Xue Y, Han B. 2022. Green synthesis of silver nanoparticles using sodium alginate and tannic acid: characterization and anti-S. aureus activity. Int J Biol Macromol 195:515–522. doi:10.1016/j.ijbiomac.2021.12.031 34920064

[23] Zang W, Cao H, Ge J, Zhao D. 2024. Structures, physical properties and antibacterial activity of silver nanoparticles of Lactiplantibacillus plantarum exopolysaccharide. Int J Biol Macromol 263:130083. doi:10.1016/j.ijbiomac.2024.130083 38423905

[24] Xiao R, Grinstaff MW. 2017. Chemical synthesis of polysaccharides and polysaccharide mimetics. Prog Polym Sci 74:78–116. doi:10.1016/j.progpolymsci.2017.07.009

[25] Tang L, Sun Y, Ge P, Chen L, Cheung PCK, Ding Z, Fang J. 2022. Biogenetic nanocarriers with enhanced pH stability formed by zein and selectively depolymerized mushroom hyperbranched β-glucans. Int J Biol Macromol 209:1771–1783. doi:10.1016/j.ijbiomac.2022.04.147 35472365

[26] Tian D, Qiao Y, Peng Q, Zhang Y, Gong Y, Shi L, Xiong X, He M, Xu X, Shi B. 2023. A poly-D-mannose synthesized by a one-pot method exhibits anti-biofilm, antioxidant, and anti-inflammatory properties in vitro. Antioxidants (Basel) 12:1579. doi:10.3390/antiox12081579 37627574 PMC10451989

[27] Cui Y, Liu X, Li S, Hao L, Du J, Gao D, Kang Q, Lu J. 2018. Extraction, characterization and biological activity of sulfated polysaccharides from seaweed Dictyopteris divaricata. Int J Biol Macromol 117:256–263. doi:10.1016/j.ijbiomac.2018.05.134 29792963

[28] Jia R-B, Li Z-R, Wu J, Ou Z-R, Zhu Q, Sun B, Lin L, Zhao M. 2020. Physicochemical properties of polysaccharide fractions from Sargassum fusiforme and their hypoglycemic and hypolipidemic activities in type 2 diabetic rats. Int J Biol Macromol 147:428–438. doi:10.1016/j.ijbiomac.2019.12.243 31899245

[29] Gu J, Zhang H, Yao H, Zhou J, Duan Y, Ma H. 2020. Comparison of characterization, antioxidant and immunological activities of three polysaccharides from Sagittaria sagittifolia L. Carbohydr Polym 235:115939. doi:10.1016/j.carbpol.2020.115939 32122481

[30] Yan J-K, Ding Z-C, Gao X, Wang Y-Y, Yang Y, Wu D, Zhang H-N. 2018. Comparative study of physicochemical properties and bioactivity of Hericium erinaceus polysaccharides at different solvent extractions. Carbohydr Polym 193:373–382. doi:10.1016/j.carbpol.2018.04.019 29773393

[31] Yan J-K, Wu L-X, Qiao Z-R, Cai W-D, Ma H. 2019. Effect of different drying methods on the product quality and bioactive polysaccharides of bitter gourd (Momordica charantia L.) slices. Food Chem 271:588–596. doi:10.1016/j.foodchem.2018.08.012 30236720

[32] Luo L, Wu Y, Liu C, Huang L, Zou Y, Shen Y, Lin Q. 2019. Designing soluble soybean polysaccharides-based nanoparticles to improve sustained antimicrobial activity of nisin. Carbohydr Polym 225:115251. doi:10.1016/j.carbpol.2019.115251 31521298

[33] Jing Y, Cheng W, Ma Y, Zhang Y, Li M, Zheng Y, Zhang D, Wu L. 2022. Structural characterization, antioxidant and antibacterial activities of a novel polysaccharide from Zingiber officinale and its application in synthesis of silver nanoparticles. Front Nutr 9:917094. doi:10.3389/fnut.2022.917094 35719161 PMC9204034

[34] Zhang L, Zhang Y, Li W, Zhou Y, Guo R, Pei X, Xie J. 2025. Chitosan and its functional derivatives for nutraceutical delivery: focus on quaternized, hydrochloride, and carboxymethyl forms. Trends Food Sci Technol 164:105237. doi:10.1016/j.tifs.2025.105237

[35] Wang HC, Chen XQ, Wen YS, Li DZ, Sun XY, Liu ZW, Yan HQ, Lin Q. 2022. A study on the correlation between the oxidation degree of oxidized sodium alginate on its degradability and gelation. Polymers (Basel) 14:1679. doi:10.3390/polym14091679 35566849 PMC9104389

[36] Gomez CG, Rinaudo M, Villar MA. 2007. Oxidation of sodium alginate and characterization of the oxidized derivatives. Carbohydr Polym 67:296–304. doi:10.1016/j.carbpol.2006.05.025

[37] Chorfa N, Nlandu H, Belkacemi K, Hamoudi S. 2022. Physical and enzymatic hydrolysis modifications of potato starch granules. Polymers (Basel) 14:2027. doi:10.3390/polym14102027 35631908 PMC9143340

[38] Raji V, Chakraborty M, Parikh PA. 2012. Synthesis of starch-stabilized silver nanoparticles and their antimicrobial activity. Part Sci Technol 30:565–577. doi:10.1080/02726351.2011.626510

[39] Vigneshwaran N, Nachane RP, Balasubramanya RH, Varadarajan PV. 2006. A novel one-pot 'green' synthesis of stable silver nanoparticles using soluble starch. Carbohydr Res 341:2012–2018. doi:10.1016/j.carres.2006.04.042 16716274

[40] Zahran MK, Ahmed HB, El-Rafie MH. 2014. Alginate mediate for synthesis controllable sized AgNPs. Carbohydr Polym 111:10–17. doi:10.1016/j.carbpol.2014.03.029 25037323

[41] Cinteza LO, Scomoroscenco C, Voicu SN, Nistor CL, Nitu SG, Trica B, Jecu ML, Petcu C. 2018. Chitosan-stabilized Ag nanoparticles with superior biocompatibility and their synergistic antibacterial effect in mixtures with essential oils. Nanomaterials (Basel) 8:16. doi:10.3390/nano8100826 PMC6215195 30322127

[42] Ma Z, Liu J, Liu Y, Zheng X, Tang K. 2021. Green synthesis of silver nanoparticles using soluble soybean polysaccharide and their application in antibacterial coatings. Int J Biol Macromol 166:567–577. doi:10.1016/j.ijbiomac.2020.10.214 33144252

[43] Raghavendra GM, Jung J, Kim D, Seo J. 2016. Step-reduced synthesis of starch-silver nanoparticles. Int J Biol Macromol 86:126–128. doi:10.1016/j.ijbiomac.2016.01.057 26802247

[44] Wei D, Sun W, Qian W, Ye Y, Ma X. 2009. The synthesis of chitosan-based silver nanoparticles and their antibacterial activity. Carbohydr Res 344:2375–2382. doi:10.1016/j.carres.2009.09.001 19800053

[45] Suleman Ismail Abdalla S, Katas H, Chan JY, Ganasan P, Azmi F, Fauzi Mh Busra M. 2020. Antimicrobial activity of multifaceted lactoferrin or graphene oxide functionalized silver nanocomposites biosynthesized using mushroom waste and chitosan. RSC Adv 10:4969–4983. doi:10.1039/c9ra08680c 35498291 PMC9049173

[46] Fissan H, Ristig S, Kaminski H, Asbach C, Epple M. 2014. Comparison of different characterization methods for nanoparticle dispersions before and after aerosolization. Anal Methods 6:7324–7334. doi:10.1039/c4ay01203h 40883942

[47] Katas H, Mohd Akhmar MA, Suleman Ismail Abdalla S. 2021. Biosynthesized silver nanoparticles loaded in gelatine hydrogel for a natural antibacterial and anti-biofilm wound dressing. J Bioact Compat Polym 36:111–123. doi:10.1177/0883911520988303

[48] Yin IX, Zhang J, Zhao IS, Mei ML, Li Q, Chu CH. 2020. The antibacterial mechanism of silver nanoparticles and its application in dentistry. Int J Nanomedicine 15:2555–2562. doi:10.2147/IJN.S246764 32368040 PMC7174845

[49] Tester RF, Al-Ghazzewi FH. 2013. Mannans and health, with a special focus on glucomannans. Food Res Int 50:384–391. doi:10.1016/j.foodres.2012.10.037

[50] Tian D, Qiao Y, Peng Q, Xu X, Shi B. 2024. Anti-biofilm mechanism of a synthetical low molecular weight poly-d-mannose on Salmonella Typhimurium. Microb Pathog 187:106515. doi:10.1016/j.micpath.2023.106515 38160987

[51] Das J, Paul Das M, Velusamy P. 2013. Sesbania grandiflora leaf extract mediated green synthesis of antibacterial silver nanoparticles against selected human pathogens. Spectrochim Acta A Mol Biomol Spectrosc 104:265–270. doi:10.1016/j.saa.2012.11.075 23270884

[52] Manikprabhu D, Lingappa K. 2013. Antibacterial activity of silver nanoparticles against methicillin-resistant Staphylococcus aureus synthesized using model Streptomyces sp. pigment by photo-irradiation method. J Pharm Res 6:255–260. doi:10.1016/j.jopr.2013.01.022

[53] Chen C-W, Hsu C-Y, Lai S-M, Syu W-J, Wang T-Y, Lai P-S. 2014. Metal nanobullets for multidrug resistant bacteria and biofilms. Adv Drug Deliv Rev 78:88–104. doi:10.1016/j.addr.2014.08.004 25138828

[54] Sondi I, Salopek-Sondi B. 2004. Silver nanoparticles as antimicrobial agent: a case study on E. coli as a model for Gram-negative bacteria. J Colloid Interface Sci 275:177–182. doi:10.1016/j.jcis.2004.02.012 15158396

[55] Qayyum S, Khan AU. 2016. Biofabrication of broad range antibacterial and antibiofilm silver nanoparticles. IET Nanobiotechnol 10:349–357. doi:10.1049/iet-nbt.2015.0091 27676385 PMC8676207

[56] Mohanty S, Mishra S, Jena P, Jacob B, Sarkar B, Sonawane A. 2012. An investigation on the antibacterial, cytotoxic, and antibiofilm efficacy of starch-stabilized silver nanoparticles. Nanomedicine 8:916–924. doi:10.1016/j.nano.2011.11.007 22115597

[57] Patel M, Kikani T, Saren U, Thakore S. 2024. Bactericidal, anti-biofilm, anti-oxidant potency and catalytic property of silver nanoparticles embedded into functionalised chitosan gel. Int J Biol Macromol 262:129968. doi:10.1016/j.ijbiomac.2024.129968 38320641

[58] Jiang W-Y, Ran S-Y. 2018. Two-stage DNA compaction induced by silver ions suggests a cooperative binding mechanism. J Chem Phys 148:205102. doi:10.1063/1.5025348 29865834

[59] Shah S, Gaikwad S, Nagar S, Kulshrestha S, Vaidya V, Nawani N, Pawar S. 2019. Biofilm inhibition and anti-quorum sensing activity of phytosynthesized silver nanoparticles against the nosocomial pathogen Pseudomonas aeruginosa. Biofouling 35:34–49. doi:10.1080/08927014.2018.1563686 30727758

[60] Huma Z-E, Javed I, Zhang Z, Bilal H, Sun Y, Hussain SZ, Davis TP, Otzen DE, Landersdorfer CB, Ding F, Hussain I, Ke PC. 2020. Nanosilver mitigates biofilm formation via FapC amyloidosis inhibition. Small 16:e1906674. doi:10.1002/smll.201906674 31984626 PMC7260094

[61] Zhang Y, Pan X, Liao S, Jiang C, Wang L, Tang Y, Wu G, Dai G, Chen L. 2020. Quantitative proteomics reveals the mechanism of silver nanoparticles against multidrug-resistant Pseudomonas aeruginosa biofilms. J Proteome Res 19:3109–3122. doi:10.1021/acs.jproteome.0c00114 32567865

[62] Chakraborty A, Biswas A. 2020. Structure, stability and chaperone function of Mycobacterium leprae heat shock protein 18 are differentially affected upon interaction with gold and silver nanoparticles. Int J Biol Macromol 152:250–260. doi:10.1016/j.ijbiomac.2020.02.182 32084461

[63] Jena P, Bhattacharya M, Bhattacharjee G, Satpati B, Mukherjee P, Senapati D, Srinivasan R. 2020. Bimetallic gold–silver nanoparticles mediate bacterial killing by disrupting the actin cytoskeleton MreB. Nanoscale 12:3731–3749. doi:10.1039/c9nr10700b 31993609

[64] Dunsing V, Irmscher T, Barbirz S, Chiantia S. 2019. Purely polysaccharide-based biofilm matrix provides size-selective diffusion barriers for nanoparticles and bacteriophages. Biomacromolecules 20:3842–3854. doi:10.1021/acs.biomac.9b00938 31478651

[65] Kalishwaralal K, BarathManiKanth S, Pandian SRK, Deepak V, Gurunathan S. 2010. Silver nanoparticles impede the biofilm formation by Pseudomonas aeruginosa and Staphylococcus epidermidis. Colloids Surf B Biointerfaces 79:340–344. doi:10.1016/j.colsurfb.2010.04.014 20493674

